# Ecthyma gangrenosum on the face of a malnourished child with *Pseudomonas* sepsis: Simulating Cancrum oris

**DOI:** 10.4102/ajlm.v7i1.756

**Published:** 2018-12-05

**Authors:** Khadijat O. Isezuo, Usman M. Sani, Usman M. Waziri, Bilkisu I. Garba, Yahaya Mohammed, Joy F. Legbo, Nazish P. Aquil, Fatima I. Abubakar, Memuna Omar

**Affiliations:** 1 Department of Paediatrics, Usmanu Danfodiyo University Teaching Hospital, Sokoto, Nigeria; 2 Department of Medical Microbiology and Parasitology, Usmanu Danfodiyo University Teaching Hospital, Sokoto, Nigeria; 3 Department of Surgery, Usmanu Danfodiyo University Teaching Hospital, Sokoto, Nigeria

## Abstract

**Introduction:**

Ecthyma gangrenosum (EG) is a cutaneous lesion commonly caused by *Pseudomonas aeruginosa* that involves mainly the lower limbs and gluteal region, seen more in immunosuppressed patients with neutropenia. Cancrum oris (Noma) is a gangrenous necrosis of the face that begins as a gingival ulcer and progresses rapidly to destroy contiguous tissues in malnourished children.

**Case Presentation:**

This article reports a case of facial EG which was similar to Noma in a malnourished child: a 16-month old girl with fever, cough, weight loss, watery stool and swelling on right cheek. She was febrile, pale, wasted with bilateral pitting pedal oedema. She had a solitary circumscribed round necrotic lesion, with surrounding hyperaemia on the right malar area which extended to destroy the right ala nasi. No intra-oral rashes but she had left ear discharge. She received blood transfusion, antibiotics, antiseptic wound care and nutritional rehabilitation.

**Management and Outcome:**

Swabs of the lesion and ear discharge both revealed Gram-negative bacilli and culture yielded *P. aeruginosa*. Retroviral, Mantoux and Gene Xpert tests were negative. She had moderate anaemia, normal white blood cell count, and neutropaenia. Parenteral ceftriazone was changed to ciprofloxacin based on sensitivity results and lack of clinical response. The wound healed with residual scarring and partial destruction of right ala nasi.

**Discussion:**

Although this patient had facial necrosis to suggest Noma, she did not have initial oral involvement, and clinical features such as *Pseudomonas* sepsis and neutropaenia suggested EG. Facial necrosis in malnourished children may be due to EG.

## Introduction

Ecthyma gangrenosum (EG) is a cutaneous lesion commonly caused by *Pseudomonas aeruginosa*,^1,2^ though it has been associated with other bacterial, viral and fungal aetiological agents.^3,4,5^ It occurs commonly in immunosuppression of various causes and is associated with neutropaenia.^6^ It may occur as a primary skin lesion which is the localised or non-septicaemic form or it may be the classical form which is a septicaemic illness associated with haematogenous spread of the causative bacterial organism.^5,7^

The typical appearance is that of a necrotic skin lesion with a central eschar surrounded by a hyperaemic halo.^8^ It commonly involves the lower limbs or genital area but may also involve other areas of the body occurring as solitary or multiple lesions. The sites commonly affected by EG lesions are the gluteal or perineal region (57%), extremities (30%), trunk (6%), and face (6%).^9^ Some have reported facial involvement in 12% of cases.^4^ Facial involvement has been reported to be common in neonates who are immunocompromised compared to older infants and children.^10,11,12^

Cancrum oris (Noma) is a severe oro-facial gangrene which occurs more in debilitated and malnourished patients.^13^ It usually starts as a gingival infected ulcer which rapidly becomes necrotic and spreads to produce extensive destruction of the tissues of the face in and around the oral cavity. Aetiology is usually polymicrobial by opportunistic organisms related to malnutrition and immune dysfunction.^14^ Some writers have described Noma-like necro-ulcerative lesions involving the face for which, if diagnosed early, intensive therapy may limit progression and confer better prognosis.^15^

In this report, we highlight a case of a malnourished child who presented with a necrotic ulcer involving the face which was similar to Noma but, on further review of the evolution of the ulcer and investigation of test results, was found more likely to be EG. We report the case to highlight that a necrotic facial ulcer presenting in a malnourished child may be EG and may mimic orofacial gangrene of Noma if not aggressively managed.

## Case presentation

### Ethical considerations

Ethical approval for the study was obtained from the Research and Ethics Committee of Usmanu Danfodiyo University Teaching Hospital (Number: UDUTH/HREC/2018/699). Written consent was obtained from the parents of the child. Confidentiality was maintained as well as privacy, as the identity of the child was known only to the authors who partook in her management. The clinical pictures taken were selectively occluded except for the aspect showing the lesion so as to prevent recognition of the patient.

### Method of data collection

Data was collected by history-taking, clinical examination, clinical photographs and anthropometric measurements (weight, height and occipitofrontal circumference); venous blood samples were collected via aseptic technique for electrolytes, complete blood count, retroviral screening and blood culture. A nasogastric tube was used to collect samples of gastric washings. A sterile swab stick was used to collect samples of the lesion and ear discharge.

### Case report

A 16-month old girl presented to Usmanu Danfodiyo University Teaching Hospital, Sokoto, in October 2016 with complaints of fever, cough and weight loss of 2 months, watery stool of 6 weeks, swelling on right cheek of a week’s duration. The swelling initially started as a reddish rash which increased in size and darkened in colour. It was painless and not associated with discharge. She had poor nutritional and immunisation history. There was no preceding history of measles, though there was contact with other children who had pertussis.

**FIGURE 1:**
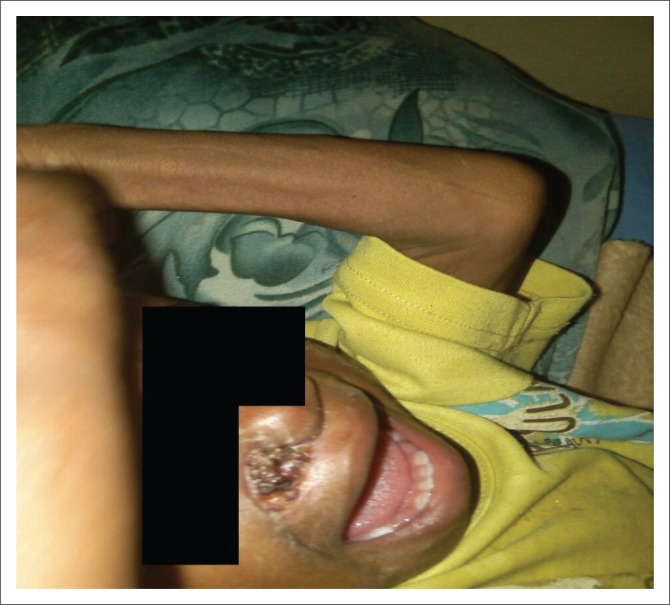
Necrotic eschar on second day after admission.

**FIGURE 2:**
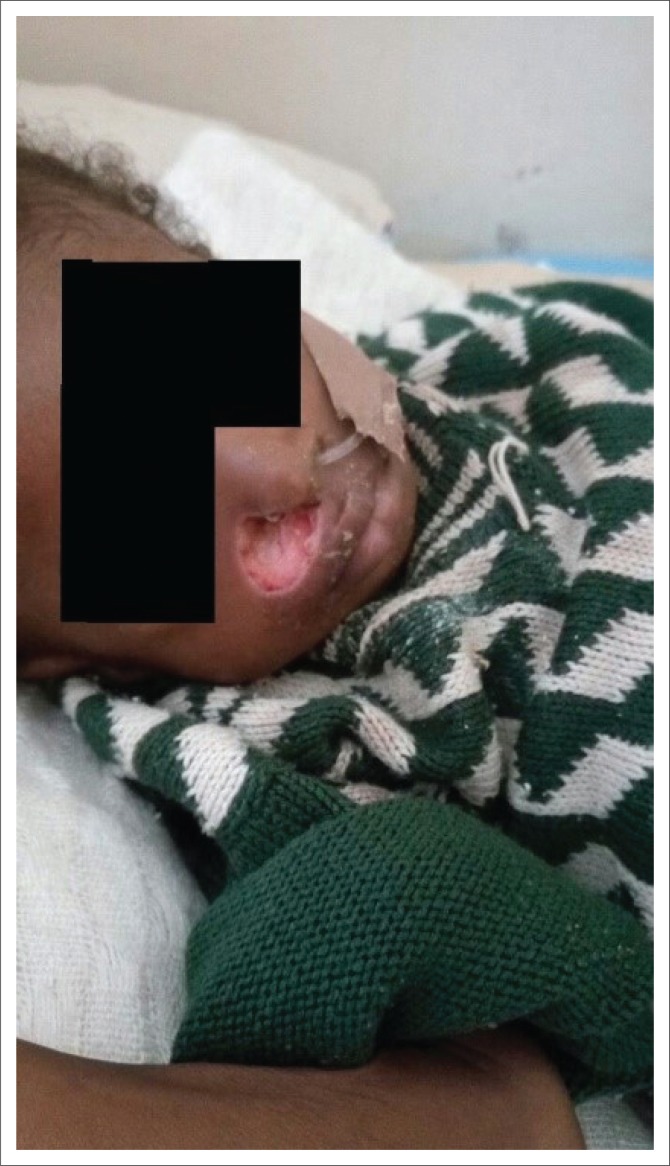
Deep ulcer extending to destroy the right ala nasi after sloughing of necrotic eschar.

On examination, she was febrile (38.9 °C), pale, wasted with bilateral pitting pedal oedema and fluffy hair. Her weight was 5 kilograms (kg), length was 68 cm (Weight for length Z-score *of* < −4 standard deviation, expected value of −2 standard deviation to +2 standard deviation), occipitofrontal circumference was 40 cm (expected range of 47 – 48 cm) and mid-arm circumference was 9.5 cm (expected normal value of >14 cm). She had a solitary circumscribed round necrotic lesion (2 cm × 2 cm) with a central black eschar and surrounding hyperaemia on the right malar area which had minimal purulent discharge but was not foul-smelling (Figure 1). The central eschar sloughed off on the third day after admission to reveal a deep ulcer which had extended to destroy the right ala nasi (Figure 2). There was no intra-oral communication of the ulcer from the external surface. She did not have any intra-oral rashes, gingival ulcers or oral thrush.

She developed purulent left ear discharge four days after admission. The external ear was normal; however, examination of the middle ear was not done as the child was not cooperative. Her pulse was 124 beats per minute and respiratory rate was 46 cycles per minute. She had vesicular breath sounds with reduced intensity and normal heart sounds. Abdominal examination revealed a non-tender hepatomegaly of 4 cm. Neurological examination was normal.

## Management and outcome

Swabs of the lesion and ear discharge showed pus cells and Gram-negative bacilli, while culture of both yielded *P. aeruginosa*. It was sensitive to ciprofloxacin^+++^, ceftazidime^++^, ofloxacin^++^, and gentamicin^++^. Blood culture was however negative, although anaerobic culture was not done. Retroviral screening, Mantoux and Gene Xpert tests were all negative. Her packed cell volume was 22%, which dropped to 18% on the seventh day, on account of which she was transfused packed red cells, resulting in post-transfusion volume of 32%. Her total white cell count was 7 × 10^9^/L (reference range 4–11 × 10^9^/L), with 16% constituting neutrophils (1120 cells, range: 2000 – 8000 cells), and 84% lymphocytes (5880 cells, range: 1000 – 6000 cells); platelets were 145 × 10^9^/L. The erythrocyte sediment rate was 28 mm fall/hr. The diagnosis was severe acute malnutrition (oedematous type) with complications of sepsis, severe anaemia and possibly evolving Cancrum oris. However, after further review of the patient’s history, evolution of the lesion, in conjunction with the investigation results suggesting neutropaenia and isolation of *P. aeruginosa* from the lesion, EG involving the face was considered to be more likely.

She received blood transfusion, antibiotics, antiseptic wound care with normal saline soaks and dressing with honey. She also received nutritional rehabilitation in form of enriched pap and ready-to-use therapeutic food which is a nutritional rehabilitation diet manufactured mainly from peanuts. Electrolyte derangements (hypokalaemia and hyponatremia) were corrected with oral potassium supplements added to feeds and oral rehydration solution. Initial intravenous ceftriazone was changed to intravenous ciprofloxacin and intravenous gentamicin based on sensitivity results.

**FIGURE 3:**
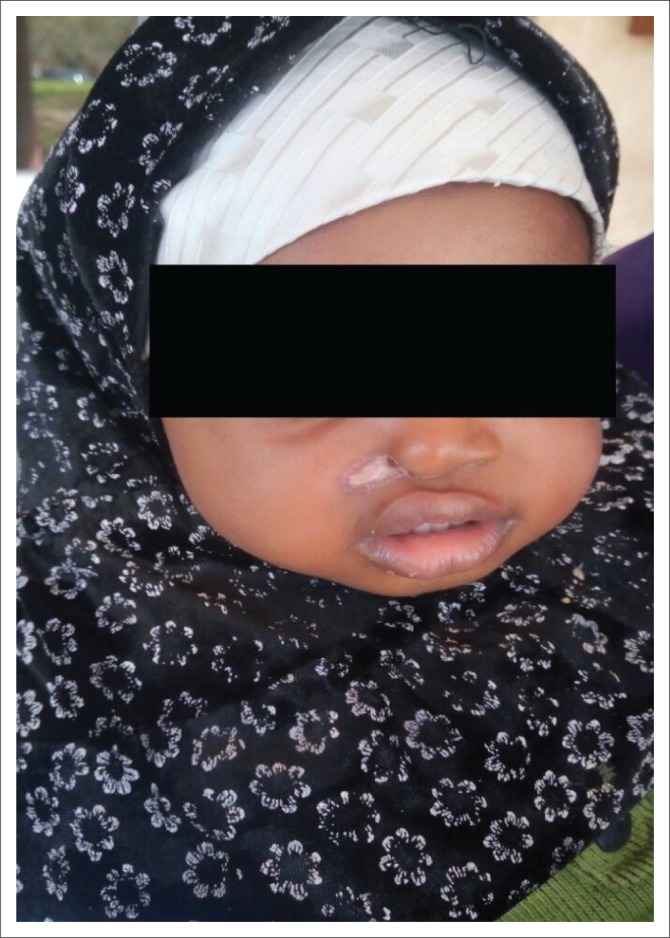
Residual scarring after healing of the ulcer.

The ulcer healed remarkably but with residual scarring, including destruction of right ala nasi (Figure 3). Oedema subsided four days after admission and her weight rose gradually during the subsequent 2 weeks to 6.8 kg. She spent about 3 weeks in the hospital. On a subsequent follow-up visit, she was doing well; however, the residual scar was still present, though much smaller. Caregivers defaulted on an appointment to see the co-managing plastic surgeon for possible repair of the residual scar.

## Discussion

Ecthyma gangrenosum lesions were first described in 1897 in association with *Pseudomonas* septicaemia.^16^ EG usually begins as a haemorrhagic vesicle or pustule that gradually evolves into a necrotic ulcer with eschar. This progression is because the bacteria invades the walls of the dermal and sub-dermal vessels leading to vascular damage and interruption of blood supply to the skin causing necrosis of the skin with eschar and ulcer formation.^17^ Although it has been recognised to be caused by a variety of other organisms, EG is most typically associated with severe systemic infection due to *P. aeruginosa*.^17,18^ It is most often seen in immunocompromised or neutropenic patients, though it can also occur in previously healthy individuals.

Our patient was immunocompromised as she was severely malnourished and she also had neutropaenia.^19,20^ The presence of acute otitis media caused by *Pseudomonas* in the child in addition to the skin manifestation of EG supported *Pseudomonas* sepsis even though the blood culture was negative. In a case report from Brazil of eight cases of EG in children, facial involvement was less common in the series. One of the cases was also a female infant with facial involvement and malnutrition similar to our case.^21^

Authors from India reported a case of a severely malnourished child with both EG involving the limbs caused by *Pseudomonas* and Noma; they suggested that both conditions can coexist with malnutrition as an underlying predisposing factor for both.^22^ A case was reported by Biddeci^23^ from Italy in a female infant who was previously healthy. She had a facial lesion similar to our patient which healed with a residual scar after treatment with anti-pseudomonal antibiotics based on a positive skin swab. Blood culture was also negative, similar to findings in our patient.

Noma neonatorum (a distinct entity from Noma in older children) which occurs in neonates, especially those with low birth weight and those born pre-term, is a rapidly progressive gangrenous lesion involving facial structures especially the mouth, nose and eyelids. It also involves the anal region and scrotum.^24^ It is also commonly associated with *P. aeruginosa* septicaemia. Although some authors have recently questioned distinguishing Noma in neonates from EG caused by *Pseudomonas*,^11^ Noma in older children is quite a distinct entity as it starts as a gingival ulcer and the ensuing gangrene rapidly spreads through muscles exposing the bone and teeth.^13^ Also, in Noma, the aetiological agents usually isolated include anaerobes like *Prevotella intermedia, Fusobacterium nucleatum, Peptostreptococcus micros, Campylobacter* and enteric Gram-negative organisms, while EG is mainly caused by *Pseudomonas*.^13^ However, these anaerobes cannot be completely ruled out in our case since anaerobic culture was not done.

The findings in the index case suggest that the child likely had EG rather than Noma given the evolution of the ulcerative process. However, without aggressive therapy or relatively early presentation of the patient, one may hypothesise that it could have spread, further simulating Noma. In conclusion, this patient had facial necrosis to suggest Noma; however, she did not have initial oral involvement, and clinical features suggested EG, which was supported by suspected *Pseudomonas* sepsis and neutropaenia. Facial necrosis in malnourished children may be due to EG.
